# Beware detrending: Optimal preprocessing pipeline for low‐frequency fluctuation analysis

**DOI:** 10.1002/hbm.24468

**Published:** 2018-11-15

**Authors:** Michael Woletz, André Hoffmann, Martin Tik, Ronald Sladky, Rupert Lanzenberger, Simon Robinson, Christian Windischberger

**Affiliations:** ^1^ Center for Medical Physics and Biomedical Engineering Medical University of Vienna Vienna Austria; ^2^ Department of Psychiatry and Psychotherapy Medical University of Vienna Vienna Austria; ^3^ Department of Biomedical Imaging and Image‐guided Therapy Medical University of Vienna Vienna Austria

**Keywords:** ALFF, artefact reduction, detrending, fALFF, fMRI, low‐frequency fluctuation, nuisance regression, preprocessing, resting state

## Abstract

Resting‐state functional magnetic resonance imaging (rs‐fMRI) offers the possibility to assess brain function independent of explicit tasks and individual performance. This absence of explicit stimuli in rs‐fMRI makes analyses more susceptible to nonneural signal fluctuations than task‐based fMRI. Data preprocessing is a critical procedure to minimise contamination by artefacts related to motion and physiology. We herein investigate the effects of different preprocessing strategies on the amplitude of low‐frequency fluctuations (ALFFs) and its fractional counterpart, fractional ALFF (fALFF). Sixteen artefact reduction schemes based on nuisance regression are applied to data from 82 subjects acquired at 1.5 T, 30 subjects at 3 T, and 23 subjects at 7 T, respectively. In addition, we examine test–retest variance and effects of bias correction. In total, 569 data sets are included in this study. Our results show that full artefact reduction reduced test–retest variance by up to 50%. Polynomial detrending of rs‐fMRI data has a positive effect on group‐level *t*‐values for ALFF but, importantly, a negative effect for fALFF. We show that the normalisation process intrinsic to fALFF calculation causes the observed reduction and introduce a novel measure for low‐frequency fluctuations denoted as high‐frequency ALFF (hfALFF). We demonstrate that hfALFF values are not affected by the negative detrending effects seen in fALFF data. Still, highest grey matter (GM) group‐level *t*‐values were obtained for fALFF data without detrending, even when compared to an exploratory detrending approach based on autocorrelation measures. From our results, we recommend the use of full nuisance regression including polynomial detrending in ALFF data, but to refrain from using polynomial detrending in fALFF data. Such optimised preprocessing increases GM group‐level *t*‐values by up to 60%.

## 1 INTRODUCTION

Within the field of neuroimaging, resting‐state functional magnetic resonance imaging (rs‐fMRI) has recently emerged as an attractive research method for studying brain function. Rs‐fMRI is based on the analysis of low‐frequency fluctuations (LFFs) present in the blood‐oxygen‐level‐dependent (BOLD) signal which has been shown to be closely related to functional networks (Biswal, Yetkin, Haughton, & Hyde, 1995). Most of these approaches use frequency‐filtered time series for finding similarities across different brain regions. In seed‐voxel analyses, the correlation coefficient or its Fisher‐transformed counterpart are usually interpreted as representing the coupling strength between network nodes (Friston, 2011). Exploratory analysis methods such as independent component analysis (Biswal et al., 2010; Smith et al., 2013) represent important alternative approaches in the assessment of functional networks in resting‐state data.

Analysis strategies based on the amplitude of LFF (ALFF) have been proposed as means to estimate the base activity in a given region by examining the power in low‐frequency bands (Deco, Jirsa, & McIntosh, 2011). The averaged square root of power in this frequency band is called the ALFF (Zang et al., 2007). A derived quantity is its fractional counterpart, fractional ALFF (fALFF); the ALFF value divided by the sum of the square root of the power in the whole spectrum (Zou et al., 2008). Differences in both measures across patient groups have been observed (Guo et al., 2012; Han et al., 2011; Hou et al., 2012) and have even been considered as possible biomarkers for a number of diseases (Liu et al., 2013; Turner et al., 2013).

While (f)ALFF maps have been shown to be highly stable between sessions (Zuo et al., 2010), over 1 hr scans and after infusion of saline (Küblböck et al., 2014), other multisite studies have reported significant (f)ALFF variability across sites and scanner vendors, especially affecting the frontal cortex (Turner et al., 2013). Different standardisation schemes have also been shown to change (f)ALFF value distributions in the brain, particularly within regions most affected by motion (Yan, Craddock, Zuo, Zang, & Milham, 2013).

Taken together, these studies suggest that variations in the fMRI signal arising from subject motion or physiological artefacts may change LFFs in fMRI data sets, thus modifying ALFF and fALFF values. Robustness against such effects is obviously a key requirement for any application of (f)ALFF values as possible biomarkers.

Nuisance regression refers to a common approach for reducing noise from fMRI data based on linear regression. Nuisance regressors are generally obtained from either physiological recordings or the fMRI data set itself. These time courses are fitted to the data using a simple linear least‐squares algorithm and subsequently subtracted (Birn, Diamond, Smith, & Bandettini, 2006; Lund, Madsen, Sidaros, Luo, & Nichols, 2006; Weissenbacher et al., 2009). Some studies even used voxel‐specific regressors to allow for removing local nuisance estimates, for example, fluctuation artefacts that affect only some coils within a multichannel receive array (Jo, Saad, Simmons, Milbury, & Cox, 2010).

A particularly appealing option for separating neural from nonneural fluctuations is based on multiecho scanning where several images are acquired within each repetition time (TR) (Evans, Kundu, Horovitz, & Bandettini, 2015; Kundu, Inati, Evans, Luh, & Bandettini, 2012). As the BOLD contrast critically depends on the echo time (TE), fluctuations in short‐TE image signal are not (directly) related to neural processes and can thus be removed from the data set without compromising neural information.

Even without multiecho data acquisition, preprocessing can have profound effects on the final rs‐fMRI connectivity map (Murphy, Birn, Handwerker, Jones, & Bandettini, 2009; Shirer, Jiang, Price, Ng, & Greicius, 2015; Weissenbacher et al., 2009). Remarkably, only a single study has examined (f)ALFF preprocessing effects so far (Turner et al., 2013). In their sample of schizophrenic patients and controls, Turner and colleagues have shown statistically significant changes to (f)ALFF after regressing out motion and non‐grey matter (GM) signals. This highlights the importance of proper preprocessing strategies and their evaluation.

In this study, we extend their approach by adopting a sophisticated method based on principal component analysis (PCA) and examine, in unprecedented detail, the consequences of preprocessing options on ALFF and fALFF. Using resting‐state data sets obtained at 3 and 7 T, the following preprocessing options and combinations thereof are examined herein:

1. bias‐field correction
2. polynomial detrending (first and second orders)
3. white matter (WM) signal regression
4. cerebrospinal fluid (CSF) signal regression
5. realignment parameter (RP) regression

Based on the results obtained, we also introduce a novel measure for LFFs denoted as high‐frequency ALFF (hfALFF) in which normalisation is restricted to the high‐frequency spectrum only.

## 2 METHODS

### 2.1 Subjects

In order to ensure the broadest relevance of this study's results, we examined resting‐state data sets acquired at three magnetic field strengths (1.5, 3, 7 T) and with subjects over a wide range of ages.

Group 1 comprised 82 subjects (42 female, age range 19–79 years, mean/*SD* 43.3/17.0 years) scanned at 1.5 T taken from the International Consortium for Brain Mapping, (Montreal, Canada) data sets as part of the 1,000 Functional Connectomes repository (http://fcon_1000.projects.nitrc.org). Each subject was scanned three times within one session. Total of 4 out of the original 86 subjects were not included in the analysis (sub53801, sub63280, sub87217, and sub93262) due to problems in mask generation and coregistration.

Group 2 included data of 30 healthy volunteers (15 female, age range 20–30 years, mean/SD 24.4/2.4 years) acquired at 3 T available in the HNU1 data set from the Hangzhou Normal University, China, via the Consortium for Reliability and Reproducibility (CoRR) as part of the 1,000 Functional Connectomes repository (http://fcon_1000.projects.nitrc.org/indi/CoRR/html/hnu_1.html)(Zuo et al., 2014). For this data set, 10 resting‐state scans were acquired per subject over the duration of about 1 month (mean time between measurements 3.7 ± 1.2 days).

Group 3 contained 23 healthy, right‐handed subjects (10 female, age range 22–32 years, mean/*SD* 25.9/2.7 years) scanned at 7 T at the Medical University of Vienna, Austria. Subjects had no history of neurological or psychiatric disorders and conformed with regular exclusion criteria for MRI assessments. They were recruited via flyers and online platforms from the local population. All subjects gave written informed consent prior to the experiment and received financial reimbursement for their participation. The study was approved by the ethics committee of the Medical University of Vienna and procedures were performed according to the Declaration of Helsinki.

### 2.2 Data acquisition

Each data set in Group 1 consisted of a resting‐state acquisition with 128 time instances of 23 slices (matrix size: 64 × 64 voxels; in‐plane voxel size: 4 × 4 mm^2^; slice thickness: 5 mm) acquired at a TR of 2 s and an MPRAGE anatomical acquisition with 1 mm isotropic resolution (http://fcon_1000.projects.nitrc.org/fcpClassic/FcpTable.html).

Data sets in Group 2 were acquired on a GE Discovery MR750 scanner (General Electric, Boston, MA) with an eight‐channel head coil. Functional echo planar imaging (EPI) images were acquired in axial orientation with TR = 2 s, TE = 30 ms with 3.4 mm isotropic resolution for 10 min in an eyes‐open resting‐state paradigm.

For Group 3, subjects were instructed to relax, think of nothing in particular and let their minds wander while visually fixating a cross presented on a screen via a digital projector. These measurements were performed on a 7T Siemens MAGNETOM scanner (Siemens Medical, Erlangen, Germany) with a 32‐channel head coil (Nova Medical, Wilmington, MA). Functional images were acquired for 6 min using the Center for Magnetic Resonance Research (University of Minnesota) multiband gradient‐echo EPI sequence (128 × 128 px^2^, 78 slices, voxel size 1.5 × 1.5 × 1 mm^3^, 25% slice gap, multiband factor 3, 1/4 field of view shift) with TR = 1.4 s, TE = 23 ms, FA = 62°, and a GRAPPA factor of 2 (Moeller et al., 2010). For EPI distortion correction, a B_0_ field map was acquired using a 2D multigradient‐echo sequence with TR = 0.67 s, TE = 4.5/8/12.5 ms, 1.72 × 1.72 mm^2^ in‐plane resolution, 33 slices, 3 mm slice thickness (20% slice gap). Temporal phase unwrapping was performed by applying the UMPIRE algorithm (Robinson, Schodl, & Trattnig, 2014). For anatomical measurements, MPRAGE data (0.7 mm isotropic resolution) were acquired with TR = 1.98 s and TE = 3.66 ms.

### 2.3 Data preprocessing

Preprocessing for all data sets (Groups 1–3) was performed using SPM12 (http://www.fil.ion.ucl.ac.uk/spm/software/spm12/) and included motion correction and nonlinear normalisation to MNI152 space. Note that correction for geometric distortion was limited to Group 3 data (7 T) as the other data sets did not include field maps. For Group 2 data (3 T), the first five time points were removed to avoid relaxation effects; Groups 1 and 2 data sets were acquired after dummy scans. As nuisance regression cannot be used for compensating biases due to inhomogeneities in coil sensitivity or excitation fields, we employed bias correction as a separate step in or preprocessing pipeline in order to determine the influence of bias‐field correction on ALFF maps. Bias fields were estimated using the N4 bias‐field correction algorithm as implemented in ANTs (Tustison et al., 2010), while limiting the estimation process to a WM mask based on individual WM probability maps, estimated by segmenting the anatomical scans of each subject. All further analysis was repeated with and without removing the estimated bias field after the final interpolation step.

### 2.4 Nuisance regression

Each data set was processed in 16 different pipelines, using a different combination of the four nuisance regressor blocks:

- detrending (Detr)
- WM time courses
- CSF time courses
- RPs

The set of detrending nuisance regressors was intended to model any system‐related fluctuations and comprised constant, linear, and quadratic trends.

Masks for WM and CSF were generated by thresholding and eroding the respective individual tissue probability maps estimated by segmenting the anatomical scans using SPM's “New Segment” function. Time courses from all voxels within WM and CSF masks were extracted and PCA using GSL (GNU Scientific Library, https://www.gnu.org/software/gsl/) was then performed for each subject separately for WM and CSF time courses using in‐house written software. Mean and first five principal components were then computed to form the WM and CSF nuisance regression blocks, that is, six WM and six CSF regressors for each subject. WM and CSF were included since these regions can be assumed not to contain any neuronal signal of interest. In addition, WM and CSF time courses have been shown to reflect physiological artefacts from respiration and cardiac action (Behzadi, Restom, Liau, & Liu, 2007; Windischberger et al., 2002). The six RPs obtained from motion correction represented the final nuisance regression block.

All 16 combinations of these nuisance regressor blocks were used in order to determine the optimal combination. All regressors were orthogonalised prior to regression with respect to the constant, linear, and quadratic regressors using a QR decomposition (Householder, 1958) as implemented in MATLAB. The final, cleaned time courses were created by subtracting the fitted regressors from the data, thereby making the residual time series orthogonal to the regressors.

Randomised versions of all regressors were created by computing the fast Fourier transform (FFT) of each regressor, adding different random values to the phase of each frequency component followed by a subsequent inverse FFT. This was repeated 25 times, in order to get a more reliable estimate of the effects in the following analysis. For each subject and iteration, the randomised phase was kept constant for all regressors. This procedure (Prichard & Theiler, 1994) ensures that the correlation between the regressors is constant after randomisation, thereby helping to create a maximally similar set of regressors without retaining any information on the temporal location of the signals. *t*‐Values for the randomised phase data set are reported as mean over 25 repetitions.

### 2.5 Calculation of (f)ALFF

All (f)ALFF maps were calculated using an in‐house MATLAB script. Time courses were Fourier transformed using the FFT, and ALFF values were computed as the sum of the magnitudes of the low‐frequency part of the spectrum (0.01–0.1 Hz). fALFF maps were computed by dividing ALFF maps by the sum of the magnitudes of all frequencies up to the Nyquist frequency (0.25 Hz at 1.5 and 3 T and 0.36 Hz at 7 T).

All (f)ALFF maps were furthermore standardised by subject‐wise division by the mean (f)ALFF value across all in‐brain voxels. This method was proposed by Zang et al. (2007) to account for a subject‐specific baselines, similar to the methods used in positron emission tomography, and was shown to compensate the dependence of (f)ALFF on the extent of subject‐specific motion (Küblböck et al., 2014). Smoothing using a Gaussian filter with a full width at half maximum of 6 mm was applied to all maps using SPM12.

### 2.6 Group analysis

For the first assessment, paired *t* tests were performed between (f)ALFF maps with and without full nuisance regression, as implemented in SPM12, that is, linear regression at each voxel, using ordinary least squares. These paired *t* tests were calculated for data with and without bias‐field correction (ALFF maps only, fALFF maps are intrinsically bias‐field corrected). All results are reported at a family‐wise error corrected threshold significance level of 0.05 (*p* < .05, FWE_whole‐brain_).

In order to assess the optimal combination of nuisance regressors, we calculated one‐sample *t* tests for (f)ALFF maps across subjects, that is, voxelwise (f)ALFF values were averaged over the group and divided by the *SE*. We used this particular measure as it allows for straightforward assessment of intersubject variability relative to the group average. It was assumed that a successful correction increases this statistic (*t*‐values), that is, it reduces variability across subjects relative to the mean amplitude. This ratio is only meaningful for nonnegative values, a condition fulfilled by all types of (f)ALFF maps that were included in this study.

To improve the interpretability of the results, voxelwise *t*‐values of each preprocessing pipeline were transformed to percent changes relative to *t*‐values without nuisance regression. Summative analyses were limited to GM using a GM mask constructed based on the MNI template's GM probability map. For ALFF, maps without bias‐field correction were used in this kind of analysis.

An increase in the number of arbitrary, independent regressors could potentially also increase the *t*‐values. To test for this effect, we additionally performed all analyses using phase‐randomised regressors, as described above (i.e., original regressors were Fourier transformed, their phases randomised and transformed back to the temporal domain).

### 2.7 Additional low frequency correction

Preliminary results of our analysis showed marked effects of detrending, especially for fALFF maps. We therefore analysed our data sets with two approaches, additional to the standard polynomial (constant, linear and quadratic) regressors. In the first approach, we simply modified fALFF such that all frequencies below 0.01 Hz were excluded in the denominator. We denoted these fluctuation measures high‐frequency fALFF or hfALFF. They are calculated as:

(1) $$\text{hfALFF}=\frac{\text{ALFF}}{\sum_{i\left(0.01\mathrm{Hz}\right)}^{i\left(f_{\text{Nyquist}}\right)}\left|{\hat{a}}_{i}\right|}$$

where ${\hat{a}}_{i}$ is defined as the Fourier coefficient at index *i*(*f*) for frequency *f*.

In the second method, we defined low‐frequency trends via time courses based on autocorrelations similar to Friman, Borga, Lundberg, and Knutsson (2004). These time courses, called exploratory trends, were estimated for each subject by performing a canonical correlation analysis (CCA) on all non‐GM time courses (i.e., WM and CSF), and their lag‐one shifted versions (after PCA‐based dimensionality reduction to 5% of the number of samples/repetitions). All individual masks used for extracting the time courses were carefully checked to make sure that no GM voxel enter PCA. These time courses are very well suited to explaining low frequency drifts and, as a data driven method, alter frequencies that are not included in the data to a lesser degree than predefined mathematical functions. Following Friman et al. (2004), (f)ALFF maps were computed using the four exploratory detrending time courses with the highest autocorrelation (instead of the polynomial detrending) for all 16 pipelines. The only difference being that no orthogonalisation of the other regressors with respect to these time courses was performed during analysis.

In addition to group‐level *t*‐values, test–retest variability can also be used to assess data quality. For two of our three data groups, multiple resting‐state data sets are available per subject. In case of 1.5 T, three data sets were acquired in one session. For 3 T, 10 data sets acquired in 10 sessions over a period of 1 month are available for each subject. All data sets underwent the different preprocessing variants and the variance across repetitions was calculated.

## 3 RESULTS

**Figure 1:**
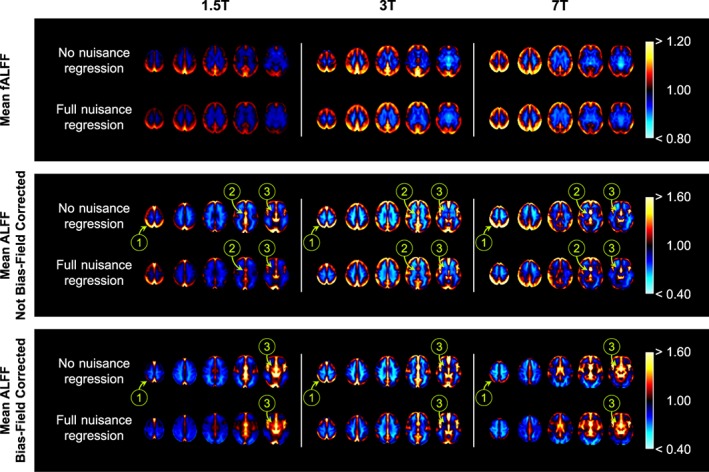
Comparison of group‐averaged (fractional) amplitude of low‐frequency fluctuation ((f)ALFF) maps with full nuisance regression versus no nuisance regression. While mean fALFF maps show little changes, more distinct changes can be seen in ALFF maps, as highlighted by the arrows (see text for details) [Color figure can be viewed at http://wileyonlinelibrary.com]

Results of the group‐averaged (f)ALFF maps obtained with full nuisance regression and without nuisance regression are depicted in Figure 1. For all field strengths, visual inspection shows only subtle differences between preprocessing approaches for fALFF and small differences for ALFF, especially if no bias‐field correction was applied. It can be seen that bias‐field correction reduces the hyperintense regions close to the MR radiofrequency coil elements (Arrow 1), especially in the more superior regions of the brain, but increases ALFF values in central brain regions (Arrow 3). This effect is more pronounced in data sets with nuisance regression. Nuisance regression also reduces high ALFF values in the ventricles (Arrow 2).

**Figure 2:**
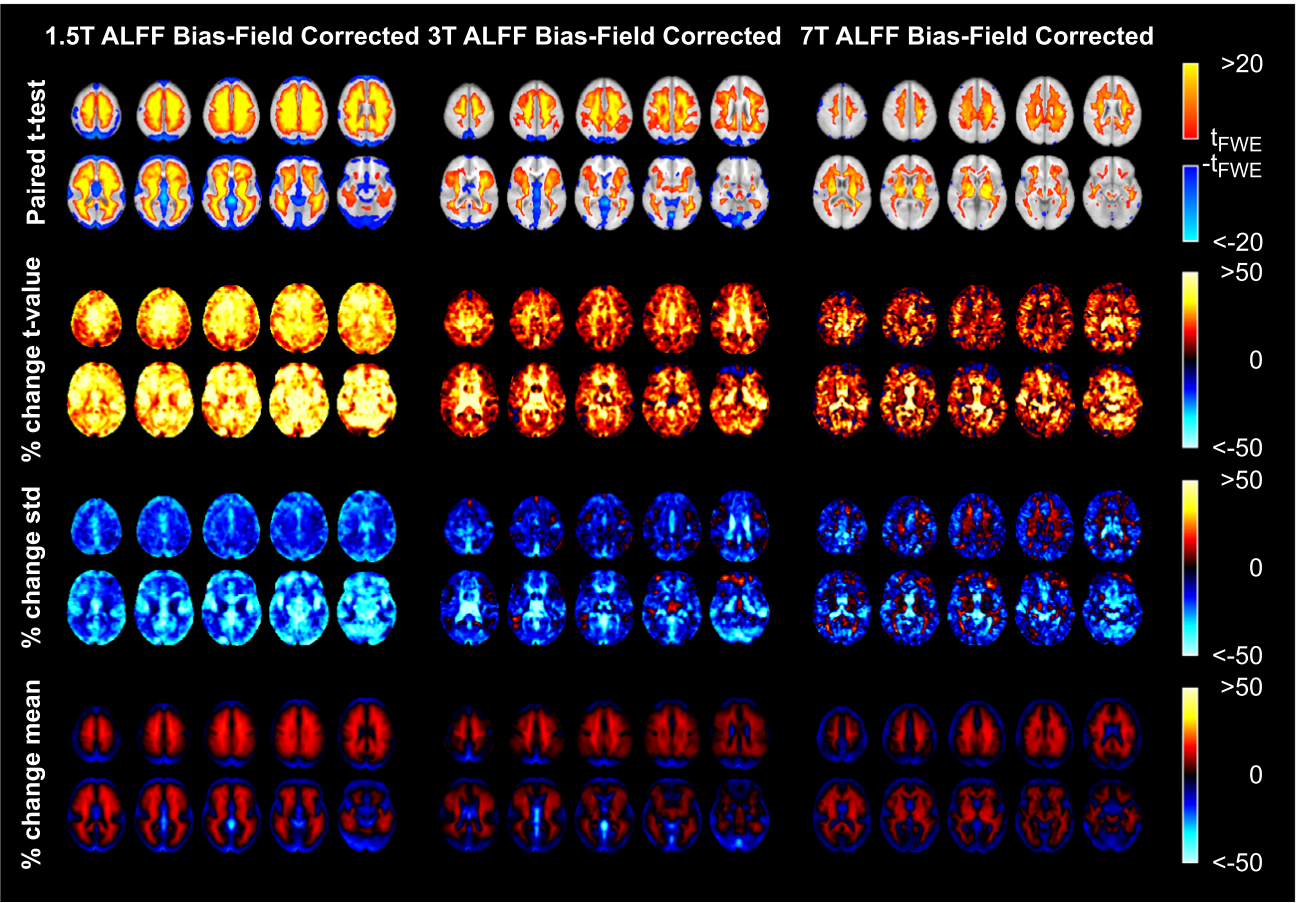
Influence of nuisance regression on bias‐corrected amplitude of low‐frequency fluctuation (ALFF) maps. Differences between ALFF maps with and without nuisance regression: Paired *t* test (*p* < .05, FWE_whole‐brain_, corresponding to 5.2, 6.2, and 7.1 for 1.5, 3, and 7 T, respectively) between individual ALFF maps (top row); relative change in group‐level *t*‐values (second row); relative change of group *SD* (third row); relative change of group mean (bottom row). The increase in *t*‐values after nuisance regression is primarily caused by a strong reduction of interindividual ALFF variance [Color figure can be viewed at http://wileyonlinelibrary.com]

Differences between bias‐corrected ALFF maps with and without nuisance regression are shown in Figure 2 (columns correspond to 1.5 T/3 T/7 T results). The top row of images show the results of paired *t* tests between individual ALFF maps and indicate an increase of ALFF in WM and subtle decrease in GM. Importantly, there is a notable increase in group‐level one‐sample *t*‐values after nuisance regression in widespread areas across the brain. Mean *t*‐value changes in GM are +45% for 1.5 T, 26% for 3 T, and + 15% for 7 T data (Figure 2, second row). As these *t*‐values were calculated by averaging ALFF maps across subjects and dividing them by the *SE*, changes could be caused by either changes in mean ALFF or changes in variance across the group. As can be seen in the third row of Figure 2, the increase in *t*‐values after nuisance regression is caused by a strong reduction of interindividual ALFF variance despite high mean ALFF values in WM (Figure 2, bottom row). ALFF maps without bias correction show similar effects (Figure S1).

**Figure 3:**
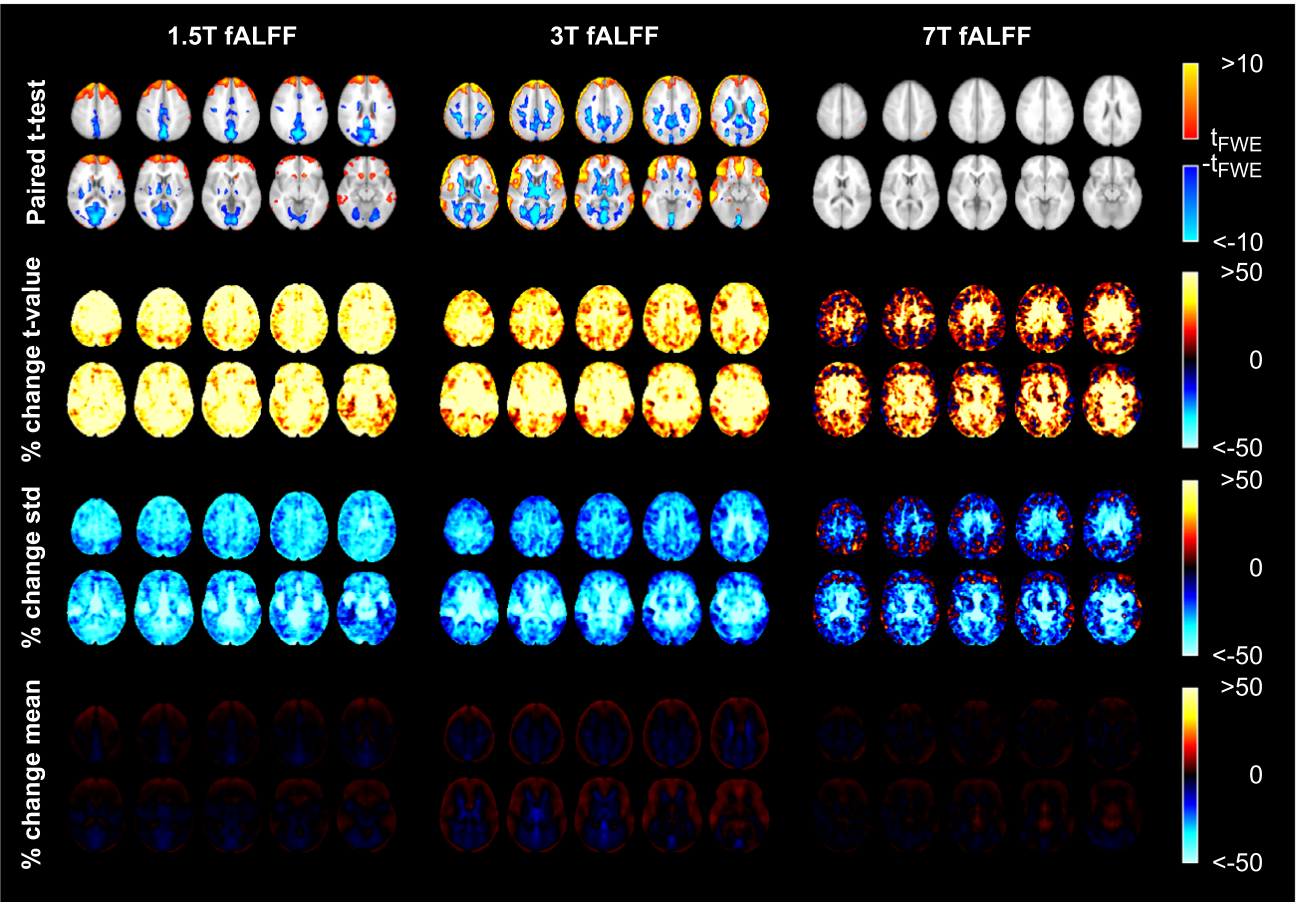
Influence of nuisance regression on fractional amplitude of low‐frequency fluctuation (fALFF) maps. Differences between fALFF maps with and without nuisance regression: Paired *t* test (*p* < .05, FWE_whole‐brain_, corresponding to 5.2, 6.1, and 7.1 for 1.5, 3, and 7 T, respectively) between individual fALFF maps (top row); relative change in group‐level *t*‐values (second row); relative change of group *SD* (third row); relative change in group mean (bottom row). While variance is reduced throughout the 1.5 T data sets, some areas of the 3 and 7 T data show increases in group variance after nuisance regression [Color figure can be viewed at http://wileyonlinelibrary.com]

In case of fALFF, pairwise *t* tests reveal some changes related to nuisance regression for 1.5 and 3 T data but not for 7 T data (Figure 3, top row). Comparing group‐level *t*‐values (Figure 3, second row) shows increases throughout the brain in 3 T data. Interestingly, some decreases in *t*‐values are apparent in GM areas of 7 T data. Closer inspection reveals that these differences are caused by changes in group‐level variance (Figure 3, third row) while mean fALFF values remain almost unchanged (Figure 3, bottom row). Averaging across all GM voxels results in *t*‐value changes of +48% for 1.5 T, 62% for 3 T, and + 22% for 7 T data. While group variance in WM is reduced after nuisance correction in all data sets, variance increases in some 7 T GM areas.

In order to analyse this unexpected increase in group variance in more detail, we examined the change in group‐wise *t*‐values for all nuisance regressor combinations. More specifically, we calculated the group‐level *t*‐value changes with and without a specific regressor (Detr, RP, WM, and CSF). These changes were then averaged for all preprocessing variants.

**Figure 4:**
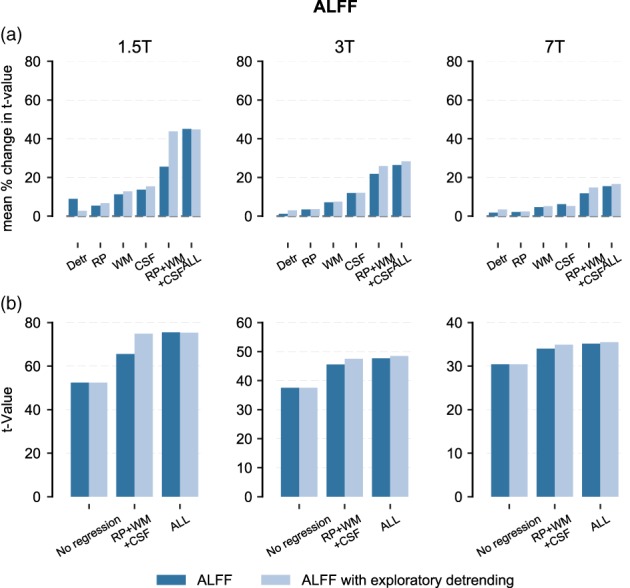
Influence of preprocessing choices on amplitude of low‐frequency fluctuation (ALFF) values. (a) Relative change in group‐level *t*‐values for the different nuisance regressors. (b) Grey matter averaged *t*‐values after nuisance regression [Color figure can be viewed at http://wileyonlinelibrary.com]

In the case of ALFF (Figure 4a, dark blue columns), group‐wise *t*‐values increased for all nuisance regressors. The highest *t*‐value increases, that is, the strongest reductions in intersubject variance, were found for the full regressor basis. Mean *t*‐value changes for detrending, RP, WM, and CSF regression were 9, 5, 11, and 14% for 1.5 T; 1, 3, and 7% for 3 T; and 2, 3, 5, and 6% for 7 T, respectively. Combining detrending, RP, WM, and CSF regression increased *t*‐values by 45, 26, and 15% for 1.5, 3, and 7 T data, respectively. Regression using phase‐randomised time courses changed group *t*‐values by 0.18 ± 0.31% across all preprocessing variants and field strengths (mean ± *SD*).

**Figure 5:**
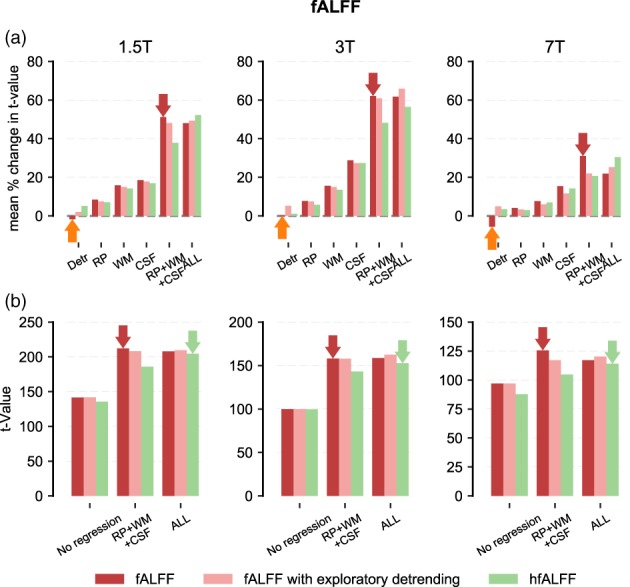
Influence of preprocessing choices on fractional amplitude of low‐frequency fluctuation (fALFF) values. Relative change in group‐level *t*‐values for the different nuisance regressors. (b) Grey matter (GM) averaged *t*‐values after nuisance regression. Polynomial detrending (Detr) reduces group‐level *t*‐values (orange arrows). Highest group‐level *t*‐values are obtained after full nuisance regression without polynomial detrending (red arrows). Green arrows indicate mean GM *t*‐values of hfALFF maps [Color figure can be viewed at http://wileyonlinelibrary.com]

In case of fALFF, however, the results are less clear (Figure 5a, red columns). RP, WM, and CSF regressions led to increases in group‐wise *t*‐values by 8, 16, and 18% for 1.5 T; 8, 16, and 29% for 3 T; and 4, 8, and 15%, for 7 T. Surprisingly, detrending (orange arrows in Figure 5) caused changes in group‐wise *t*‐values of −2 and −6% for 1.5 and 7 T, while at 3 T minimal change was observed (<0.2%). This detrimental effect of detrending was observed for fALFF but not ALFF maps indicating that the normalisation process used in fALFF calculation was the decisive factor. Preprocessing with phase‐randomised time courses changed group *t*‐values by 1.44 ± 1.87% across all regression variants and field strengths (mean ± *SD*).

Targeting the mechanism behind the effects of this normalisation step, we repeated our analysis on a new set of fALFF maps which were normalised without using the low‐frequency components, that is, the frequency range used for calculating spectral power in the denominator was limited to frequencies above 0.01 Hz. We denoted these fluctuation measures high‐frequency fALFF or hfALFF. The corresponding plots of hfALFF group‐level *t*‐value changes after detrending and WM, CSF, and RP regression are shown as green columns in Figure 5. From these results, it can be seen that—contrary to fALFF—hfALFF maps benefited from detrending (5, 1, and 4% for 1.5, 3, and 7 T, respectively). However, comparing the absolute group‐level *t*‐values between fALFF and hfALFF shows that fALFF results based on preprocessing without detrending (Figure 5b, red arrows) yielded higher numbers than hfALFF with full regression (Figure 5b, green arrows).

In addition, we also wanted to explore alternative detrending techniques that do not require a novel definition of LFF measures. To this end, we implemented exploratory detrending (described in Section 2) instead of traditional polynomial detrending. The results for these maps can be seen as pink columns in Figures 4 and 5 for ALFF and fALFF maps, respectively. While full nuisance regression using exploratory detrending led to the highest increase in ALFF *t*‐values, absolute group‐level *t*‐values for fALFF preprocessing without detrending were higher than the exploratory detrending variant.

**Figure 6:**
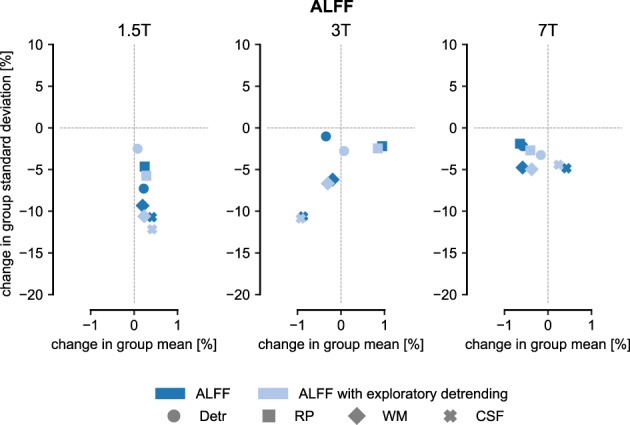
Comparison of relative changes in group‐level ALFF mean (*x* axis) and *SD* (*y* axis). While changes in mean *t*‐values are below 1%, group variance is strongly reduced by all preprocessing variants [Color figure can be viewed at http://wileyonlinelibrary.com]

**Figure 7:**
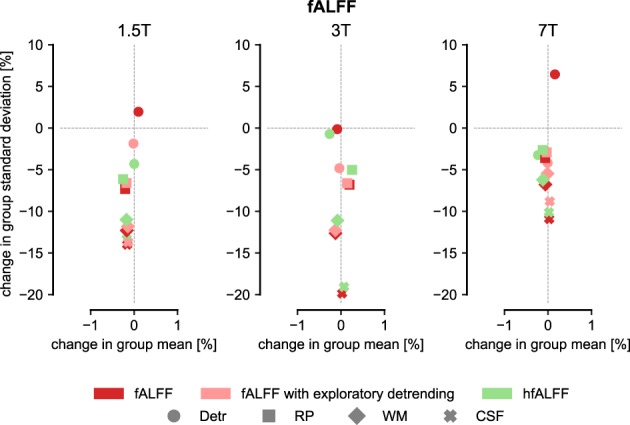
Comparison of relative changes in group‐level fractional amplitude of low‐frequency fluctuation (fALFF) mean (*x* axis) and *SD* (*y* axis). While changes in mean *t*‐values are below 1%, RP, WM, and CSF regression yield reductions of the *SD* across subjects of up to −20%. Polynomial detrending before fALFF calculation causes either no (3 T) or even an increase in group‐level variance (1.5 and 7 T) [Color figure can be viewed at http://wileyonlinelibrary.com]

In order to allow for a comprehensive assessment of preprocessing effects, we plotted changes in group‐level means and *SD* for all preprocessing variants in Figures 6 and 7. It can be seen that preprocessing had only subtle effects in group‐level means (all changes are less than 1%). Contrary, preprocessing has profound effects on variance across the group. While RP, WM, and CSF regression caused reductions in group variance of 2–20% across all field strengths, polynomial detrending of fALFF had no (3 T) or even detrimental effects of group variance (+2 and + 7% for 1.5 and 7 T, respectively).

**Figure 8:**
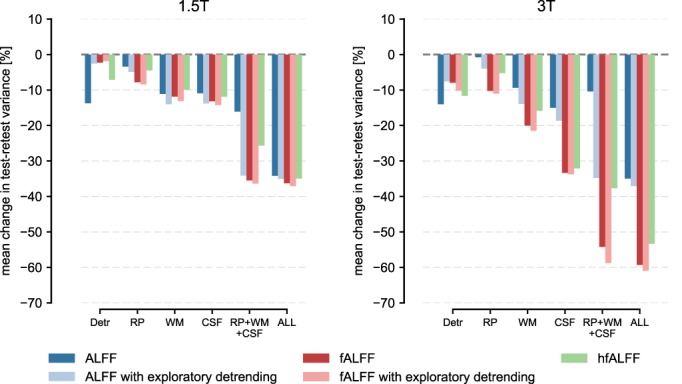
Changes in test–retest variance for the different preprocessing approaches. Results are based on three data sets per subject at 1.5 T and 10 data sets per subject at 3 T. All preprocessing variants yield reductions in test–retest variability. Strongest reductions are found for RP/WM/CSF regression and exploratory detrending of fractional amplitude of low‐frequency fluctuation (fALFF) maps. For ALFF maps, polynomial detrending is clearly indicated, whereas fALFF maps show only small differences between realignment parameter (RP)/white matter (WM)/cerebrospinal fluid (CSF) correction with and without detrending [Color figure can be viewed at http://wileyonlinelibrary.com]

For a more comprehensive assessment of the effects of different preprocessing variants, we also calculated test–retest variance across repeated resting‐state data acquisitions. Figure 8 shows the changes test–retest variance for all LFF measures (note that no test–retest data was available for 7 T data). It is apparent that all preprocessing variants yielded reductions in test–retest variance. The strongest reductions were found for preprocessing with exploratory detrending and RP/WM/CSF regression. With respect to polynomial detrending, 1.5 T data show subtle differences (<2%) between approaches with and without detrending, while 3 T data indicate stronger variance reduction when detrending is applied (−45 and −49%, respectively).

## 4 DISCUSSION

In this study, we assessed the effects of different regression‐based artefact reduction variants on (f)ALFF maps. Our results clearly show that the choice of regression approach greatly influences the (f)ALFF maps calculated.

### 4.1 Bias‐field correction

For ALFF maps, bias‐field correction of the EPIs has a strong effect, since it propagates directly into the resulting maps. At the same time, fALFF maps are intrinsically unaffected since all linear terms are cancelled during the voxelwise normalisation. In the ALFF maps, bias‐field correction reduces elevated values close to RF coils used in multichannel receive arrays (which are commonly used in fMRI studies). This can be seen as a beneficial effect as these elevated values are only related to signal reception without any physiological basis and therefore need to be corrected. Bias‐field correction as used in this study furthermore led to increased values in the centre of the brain (see Figure 1), even though image intensities in the bias‐field corrected EPIs clearly showed very homogenous white and GM intensities throughout the brain. As there is no physiological reason for elevated ALFF values close to the centre, we conclude that this effect is introduced by the bias‐field correction itself. Bias‐field correction aims to equalise spatial differences in signal amplitude, that is, after bias‐field correction the signal amplitude should be constant throughout a homogeneous object. The reduction of signal‐to‐noise ratio (SNR) with increasing distance from the RF‐coil (Wright & Wald, 1997) will consequently cause a relative increase of noise levels in regions closer to the centre of the brain. This effect increases ALFF values in low‐SNR regions after bias‐field correction since ALFF values increase with noise power. High‐SNR regions (i.e., regions close to the imaging coils) on the other hand, will benefit from bias‐field correction, since the tSNR is limited in these regions by the physiological noise, which scales with the signal (Kruger & Glover, 2001; Triantafyllou et al., 2005; Triantafyllou, Polimeni, & Wald, 2011). Correcting for signal strength will therefore appropriately correct ALFF values in these regions, without disproportionately amplifying noise. Still, any error in estimating the bias field will also linearly propagate into the resulting ALFF maps.

Normalisation of ALFF values, that is, division by the mean ALFF, can be considered standard, as it has been shown that normalisation reduces the influence of motion on ALFF values (Küblböck et al., 2014). As a consequence of this normalisation, any local changes to ALFF due to bias correction will change ALFF in all other brain voxels as well. It is important to consider this when ALFF values in different brain regions are compared between studies. On the other hand, the effects of bias‐field correction on statistical tests will be limited, since the estimated bias fields and therefore changes in ALFF maps will be rather similar across subjects, especially in regions with low gradients in the bias field as long as the same coil and setup was used for all subjects.

### 4.2 Nuisance regression

In general, nuisance regression was found to be highly beneficial in reducing intersubject variance. This is particularly apparent when examining test–retest variance. Here, our results show a reduction of test–retest variance of almost 50%.

However, our results also show that detrending using polynomial regressors should be applied with caution. In case of ALFF, the largest benefits were found for the full regression scheme, including polynomial detrending. The consistency of fALFF maps, that is, ALFF normalised to the whole frequency spectrum, was not improved by polynomial detrending. On the contrary, intersubject variance was increased by polynomial detrending. Interestingly, the results of test–retest variance comparisons do not indicate such a detrimental effect for polynomial detrending.

In an attempt to explore this unexpected result, we have introduced a novel fluctuation measure, hfALFF, which is based on normalised ALFF based on frequency power above 0.01 Hz. The fact that hfALFF maps were positively affected by polynomial detrending indicates that these very low frequencies caused the *t*‐values reductions seen in fALFF maps. As such, it may be argued that hfALFF values might be preferable to standard fALFF. We also compared the absolute group‐level *t*‐values and found that while polynomial detrending of hfALFF reduced intersubject variance, mean *t*‐values of hfALFF were still below their fALFF counterparts. Replacing fALFF by hfALFF can thus not be recommended.

Explicitly or implicitly removing low‐order polynomial trends seems to be less well suited for removing the effects of low frequency drifts in the data than the noise reduction properties inherent in the normalisation step of fALFF. Fitting and removing a regressor that contains drifts affects a wide range of frequencies. We show this issue in more detail below, where we derive an equation for least‐square fitting in the Fourier domain.

Given a signal *y* of length *N* and a nuisance regressor matrix ***x***, the linear least squares estimate can be described as:

(2) $$\beta ={\left(x^{H}x\right)}^{-1}x^{H}y$$

where ***x***^*H*^ denotes the complex conjugate transpose of *x*. The discrete Fourier transform (DFT) can be written using matrix algebra as the product of a Fourier matrix and a signal vector. The elements of the Fourier matrix are

(3) $$F_{k,n}=e^{-2\pi i\left(k-1\right)\left(n-1\right)/N}$$

For row index *k* = 1…*N* and column index *n* = 1…*N*. Without loss of generality, we can define a unitary DFT matrix *U* with the property *U*^−1^ = *U*^*H*^

(4) $$U=\frac{F}{\sqrt{N}}$$

The Fourier transforms of the signal vector *y* and the regression matrix ***x*** are

(5) Y=Uy

(6) X=Ux

Using the properties of ***U***, the linear least squares estimate can be rewritten as

(7) $$\beta ={\left(X^{H}UU^{H}X\right)}^{-1}X^{H}UU^{H}Y$$

(8) $$\beta ={\left(X^{H}X\right)}^{-1}X^{H}Y$$

Fitting the nuisance model to the data in time domain is therefore identical to fitting the data in Fourier domain. The cleaned time course $\hat{y}$ and its Fourier transformed version $\hat{Y}=U\hat{y}$ are therefore:

(9) $$\hat{y}=y-x\beta$$

(10) $$\hat{y}=y-x{\left(x^{H}x\right)}^{-1}x^{H}y$$

and

(11) $$\hat{Y}=Y-X\beta$$

(12) $$\hat{Y}=Y-X{\left(X^{H}X\right)}^{-1}X^{H}Y$$

(13) $$\hat{Y}=\left(I-X{\left(X^{H}X\right)}^{-1}X^{H}\right)Y$$

Nuisance regression can therefore be interpreted as a linear transformation of the Fourier components of the time course, where the transform solely depends on the composition of the nuisance regressor matrix in Fourier space. In this sense, it is a special kind of Fourier filter, where each frequency component is not only adapted but also combined with all other frequency components.

From these equations, it may be concluded that every frequency component may be distorted due to other, more dominant components. Importantly, if these frequencies were not present in the data, they will be introduced due to the removal of the regressor (Chen, Jahanian, & Glover, 2017; Hallquist, Hwang, & Luna, 2013). (f)ALFFs, as spectral measures, can be very sensitive to such changes and depending on whether or not further noise reduction methods are applied, group results can improve or worsen. Based on our results, we therefore cannot recommend the use of detrending for fALFF maps. It is, however, important to remove these polynomial trends from all other regressors. For ALFF maps, detrending can be recommended, since ALFF calculation does not entail inherent noise reduction properties (i.e., division by overall spectral power) and will therefore benefit from the explicit removal of these trends. Alternatively, the use of exploratory detrending (Friman et al., 2004) positively affects both fALFF and ALFF maps and can be used instead of traditional detrending. Exploratory detrending was found to yield increased group‐level *t*‐values, with absolute *t*‐values comparable to ALFF with full regression and fALFF with full regression without polynomial detrending.

However, extracting the exploratory detrending time courses from the data can be challenging. First, masks must not contain any GM voxels. Second, an additional data reduction step is necessary since in fMRI data sets usually have many more voxels than time points (Friman et al., 2004). The numbers of retained dimensions has to be estimated and will depend on the length of the time series and the data itself. If too few dimensions are retained, the data will not be very well represented and if too many are retained, the matrices in the CCA might not be of full rank.

Exploratory detrending is closely related to PCA. PCA can be used as the necessary dimensionality reduction step needed as a preprocessing step for estimating the exploratory trends. In this case, the difference between PCA and the estimation of the exploratory trends is that instead of removing the subspace of the PCA with the absolute highest variance, the subset with the highest autocorrelation is removed from the high variance subspace.

Similar to studies investigating the effects of nuisance regression for functional connectivity metrics (Murphy et al., 2009; Shirer et al., 2015; Weissenbacher et al., 2009), our results demonstrate that nuisance regression increases the consistency of (f)ALFF values across subjects. Generally, we observed an increase in *t*‐values with increasing numbers of regressors. It can be seen that for ALFF, any kind of nuisance regression increases group‐wise *t*‐values, whereas for fALFF, some kinds might even cause reductions.

Our findings show a different spatial pattern of (f)ALFF distribution before/after nuisance regression compared to Turner et al. (2013). This might be explained by the different nuisance signal models used, as our study extended the approach used by Turner and colleagues by the first principal components in the CSF and WM, making it capable of capturing more data variance.

Reducing large outliers, for example, the CSF in ALFF maps, significantly shifts the mean whole‐brain ALFF value and therefore affects all other regions as well. For ALFF, we observed the greatest changes in WM, but found less changes in the fALFF maps (see Figure 3). This effect can largely be attributed to the high‐order CSF regression (mean and five principal components), which was able to remove large parts of the CSF signal.

Our results rely on the assumption that, for healthy subjects, the removal of nuisance signals should reduce the variance in the data more than the mean. A limitation of this approach is that the lower bounds of (f)ALFF is the system noise in the images and perfect homogeneity with a mean greater than zero would therefore be achieved when any signal was removed from the time series in all subjects. Due to the fact that only the mean signal in WM was minimally affected by the regression, we are convinced that we are well above these limits and have not removed any signal of interest in our analysis.

The present study is based on data acquired at 1.5, 3, and 7 T. While ultrahigh field‐strength data are characterised by increased specificity of the BOLD effects measured (Yacoub et al., 2001), effects due to physiological noise and other artefacts are also enhanced (Triantafyllou et al., 2005). Nevertheless, our 1.5 and 3 T results clearly show that nuisance regression can be even more beneficial at field strengths lower than 7 T, as group‐level *t*‐value increases due to preprocessing were twice as high at 1.5 and 3 T than at 7 T (about 50% for 1.5 and 3 T and 25% for 7 T).

In conclusion, we have systematically assessed the influence of a number of preprocessing strategies in (f)ALFF studies and have demonstrated the need for appropriate preprocessing in order to control for nonneural, confounding factors during data acquisition and analysis. Based on test–retest reliability as a measure for data quality, we have shown that full regression will yield the strongest reduction in (f)ALFF variance across sessions. Regarding group homogeneity, we have demonstrated that nuisance regression generally increases homogeneity, with detrending being a particularly critical technique in requiring different treatment for ALFF and fALFF maps. Overall, the results of this study clearly identified data preprocessing as a crucial step in (f)ALFF calculation.

## CONFLICT OF INTERESTS

This publication reflects the views only of the authors, and the European Commission cannot be held responsible for any use that may be made of the information contained therein. The authors declare that the research was conducted in the absence of any commercial or financial relationships that could be construed as a potential conflict of interest. The authors are happy to share the code used for PCA and exploratory detrending on request.

## Supporting information


**Figure S1** Influence of nuisance regression on ALFF maps without bias‐correction. Differences between ALFF maps with and without nuisance regression: paired *t* test (*p* < .05, FWE_whole‐brain_, corresponding to 5.2, 6.2, 7.1 for 1.5 T, 3 T, 7 T, respectively) between individual ALFF maps (top row); relative change in group‐level *t*‐values (second row); relative change of group standard deviation (third row); relative change of group mean (bottom row). The increase in *t*‐values after nuisance regression is primarily caused by a strong reduction of inter‐individual ALFF variance.
