# Supplementary figures and images for: Beware detrending: Optimal preprocessing pipeline for low‐frequency fluctuation analysis
Source: Hum Brain Mapp. 2018 Nov 15;40(5):1571–82. doi: 10.1002/hbm.24468 (PMC6587723; doi:10.1002/hbm.24468)

1.5T ALFF

3T ALFF

7T ALFF

Paired t-test

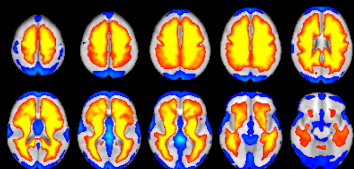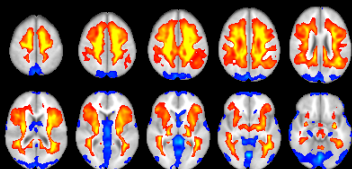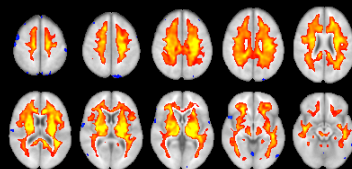 $t_{FWE}$   
 $-t_{FWE}$ 

&gt;20

&lt;-20

% change t-value

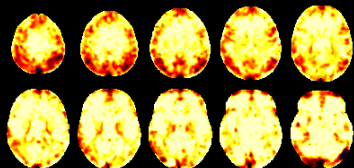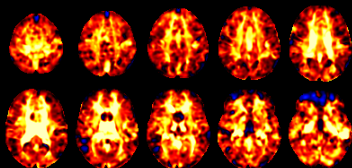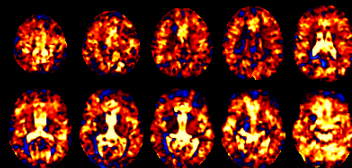

&gt;50

0

&lt;-50

% change std

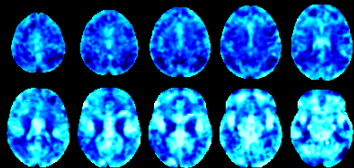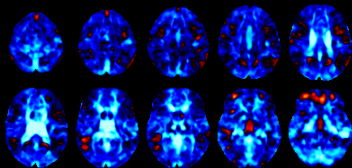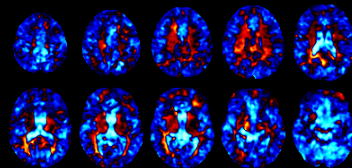

&gt;50

0

&lt;-50

% change mean

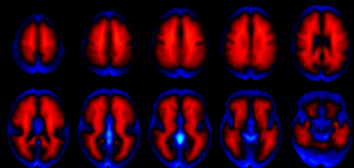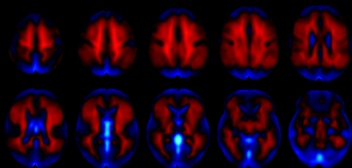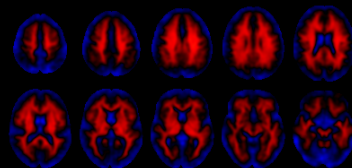

0

&lt;-50

Supplement: Supplementary file 1 — Figure S1 Influence of nuisance regression on ALFF maps without bias‐correction. Differences between ALFF maps with and without nuisance regression: paired t test (p < .05, FWEwhole‐brain, corresponding to 5.2, 6.2, 7.1 for 1.5 T, 3 T, 7 T, respectively) between individual ALFF maps (top row); relative change in group‐level t‐values (second row); relative change of group standard deviation (third row); relative change of group mean (bottom row). The increase in t‐values after nuisance regression is primarily caused by a strong reduction of inter‐individual ALFF variance. [file HBM-40-1571-s001.pdf]
